# Clinical and pathological features of *BRCA1* associated carcinomas in a hospital-based sample of Dutch breast cancer patients

**DOI:** 10.1054/bjoc.2001.2103

**Published:** 2001-09-01

**Authors:** G H de Bock, R A E M Tollenaar, H Papelard, C J Cornelisse, P Devilee, M J van de Vijver

**Affiliations:** Department of Medical Decision Making; Department of Surgery; Department of Pathology, Department of Human and Clinical Genetics, Leiden University Medical Centre, Leiden, The Netherlands

**Keywords:** breast neoplasms, *BRCA1* gene, population study, histology

## Abstract

Thus far, studies investigating the differences in tumour characteristics between breast cancer in *BRCA1*-carriers and other patients, have focused on highly selected groups of patients, potentially limiting the conclusions that can be drawn. Previously, we had identified 10 patients with *BRCA1* germline mutations in a hospital-based series of 642 breast cancer patients not selected for age or family history. The aim of this analysis is to investigate the clinical and pathological features of these BRCA1 associated carcinomas as compared to other breast cancers in this representative sample. Tumours from patients with *BRCA1* germline mutations (*n*= 10) were compared to an age-matched sample of other patients (*n*= 50) from the same cohort. The following characteristics were considered: axillary nodal status and tumour size, histologic parameters (tumour type, histologic grade, mitotic rate, tubule formation, nuclear grade, CIS and lymphangio invasion) and expression of several proteins (oestrogen and progesterone receptors, cyclin D1, p53, HER2/neu, E-cadherin). In *BRCA1*associated tumours receptors for oestrogen and progesterone were expressed less frequently (respectively *P*= 0.001 and *P*= 0.002) than in controls, which is in line with findings from other studies. Other differences were also in accordance with findings from other studies, although not statistically significant. We conclude that the features of *BRCA1* associated tumours detected in a hospital-based series of breast cancer patients not selected for family history of age at diagnosis are similar to tumours in cases selected for family history or age at diagnosis. © 2001 Cancer Research Campaign  http://www.bjcancer.com


*British Journal of Cancer* (2001) **85**(9), 1347–1350
© 2001 Cancer Research Campaign
doi: 10.1054/ bjoc.2001.2103, available online at http://www.idealibrary.com on IDEAL

http://www.bjcancer.com

### Clinical and pathological features of *BRCA1* associated carcinomas in a hospital-based sample of Dutch breast cancer patients

**GH de Bock[1], RAEM Tollenaar[2], H Papelard[1], CJ Cornelisse[3], P Devilee[3,4] and MJ van de Vijver[3]**

[1]Department of Medical Decision Making; [2]Department of Surgery; [3]Department of Pathology; and [4]Department of Human and Clinical Genetics, Leiden University Medical Centre, Leiden, The Netherlands

**Summary** Thus far, studies investigating the differences in tumour characteristics between breast cancer in *BRCA1*-carriers and other patients, have focused on highly selected groups of patients, potentially limiting the conclusions that can be drawn. Previously, we had identified 10 patients with *BRCA1* germline mutations in a hospital-based series of 642 breast cancer patients not selected for age or family history. The aim of this analysis is to investigate the clinical and pathological features of these BRCA1 associated carcinomas as compared to other breast cancers in this representative sample. Tumours from patients with *BRCA1* germline mutations ($n = 10$) were compared to an age-matched sample of other patients ($n = 50$) from the same cohort. The following characteristics were considered: axillary nodal status and tumour size, histologic parameters (tumour type, histologic grade, mitotic rate, tubule formation, nuclear grade, CIS and lymphangio invasion) and expression of several proteins (oestrogen and progesterone receptors, cyclin D1, p53, HER2/neu, E-cadherin). In *BRCA1* associated tumours receptors for oestrogen and progesterone were expressed less frequently (respectively, $P = 0.001$ and $P = 0.002$) than in controls, which is in line with findings from other studies. Other differences were also in accordance with findings from other studies, although not statistically significant. We conclude that the features of *BRCA1* associated tumours detected in a hospital-based series of breast cancer patients not selected for family history of age at diagnosis are similar to tumours in cases selected for family history or age at diagnosis.
© 2001 Cancer Research Campaign http://www.bjcancer.com

**Keywords**: breast neoplasms; *BRCA1* gene; population study; histology

Several studies have investigated the differences in tumour characteristics between breast cancer in *BRCA1*-carriers and other patients, aiming to obtain insight into features that might be of importance for recognizing patients with *BRCA1*-related tumours. In many studies, it was observed that *BRCA1*-associated breast cancers are more frequently histologic grade 3 (Eisinger et al, 1996; Marcus et al, 1996; Johannsson et al, 1997; Karp et al, 1997; Lakhani et al, 1997; Armes et al, 1998; Lynch et al, 1998) and that they express receptors for oestrogen and progesterone less frequently (Johannsson et al, 1997; Karp et al, 1997; Lynch et al, 1998; Loman et al, 1998 Verhoog et al, 1998; Armes et al, 1999). Both features are usually associated with a poorer prognosis.

However, all studies were performed on highly selected groups of patients, which might lead to survival bias and underestimation of the differences under study. The majority of studies examined tumours from families with a high incidence of breast cancer (Eisinger et al, 1996; Marcus et al, 1996; Johannsson et al, 1997; Lakhani et al, 1997; Loman et al, 1998; Lynch et al, 1998;Verhoog et al, 1998), from patients with an early onset breast cancer (Armes et al, 1998, 1999), or from specific populations with founder mutations (Karp et al, 1997). Moreover, in most studies in which the cases were selected from breast cancer families, the controls were breast cancer patients without a positive family history but no definite proof of a negative *BRCA1* mutations status (Eisinger et al, 1996; Verhoog et al, 1998) or an unknown family history (Marcus et al, 1996; Johannsson et al, 1997; Lakhani et al, 1997). This might also give an underestimation of the differences under study.

Recently, we completed a *BRCA1* mutation screening study on a hospital-based cohort of 642 breast cancer patients not selected for family history or age at diagnosis. *BRCA1*-germline mutations were identified in 10 patients by using a mutation-scanning assay that would detect approximately 70% of the currently known Dutch mutation spectrum (Papelard et al, 2000). The aim of this analysis is to investigate the clinical and pathological features of these *BRCA1* associated carcinomas as compared to other breast cancers in a representative sample.

### METHODS

### Patients and Setting

#### *Population*

From January 1984 until November 1996 all primary invasive breast cancer patients surgically treated at the Leiden University Medical Centre (LUMC) regardless of age or family history have been asked to provide a blood sample for research purposes. Mutation screening of *BRCA1* was performed anonymously using an assay targeted at known founder mutations in the Dutch population. In brief, 15 PCR-amplifiers covered gene regions known to

*Received 17 January 2001*
*Revised 26 July 2001*
*Accepted 7 August 2001*

*Correspondence to:* GH de Bock contain a clustering of small deletions and insertions. PCR-products were size-fractionated on an ABI377™ DNA sequencer, and those showing additional bands were re-amplified and sequenced. In addition, two PCR-amplifiers specifically detected the junction of the two major large genomic deletions when present (Petrij-Bosch et al, 1997). Based on the currently known mutation incidence among Dutch breast cancer families (http://www.medfac.leidenuniv.nl/lab-devilee/Lab/b1nlmuts.htm), this assay would detect ~70% of that incidence. The Committee of Medical Ethics of the LUMC approved the study protocol. A more detailed description of this population and the BRCA1 mutation testing is given in Papelard et al, 2000.

### *Patients*

For the current analysis, tumours of patients with *BRCA1* associated carcinomas were compared to tumours of patients where no *BRCA1* mutation was detected. For each patients with *BRCA1* associated carcinomas five controls were selected, matched by age at diagnosis of the first breast cancer.

### Measurements

### *Clinical characteristics*

Data on age, tumour size, axillary nodal status, and preoperative therapy were collected from the medical records. Tumour size was extracted from the local pathology report.

### *Histological typing*

Histological typing was done according to the WHO classification of 1981. The invasive cancer was graded 1, 2 or 3 with the Bloom-Richardson system modified by Elston and Ellis (Elston et al, 1991). Other features assessed were type and amount of in situ carcinoma around the invasive lesion and lymphangio invasion.

### *Immunohistochemical typing*

The immunohistochemical assays were performed in one reference laboratory, using 4-μm tissue sections. Staining with each monoclonal antibody was performed in one session for the whole series. Anti-oestrogen receptor (ER) antibody (1D5, DAKO) was diluted 1:100, anti-progesterone receptor (PR) antibody (A 0098, DAKO) diluted 1:200, anti-HER2/neu antibody (3B5) (Van de Vijver, 1988) diluted 1:20 000, anti-p53 antibody (DO-7, DAKO) diluted 1:500, anticyclin D1 antibody (DCS-6, Neomarkers) diluted 1:500 and anti-E-cadherin antibody (C 20820, Transduction laboratories) diluted 1:8000. Before incubation, antigen retrieval was performed in 10 mmol/L citrate buffer for all antibodies, except the anti HER2/neu antibody (Cattoretti et al, 1993). Biotinylated rabbit-antimouse immunoglobulin and a preformed complex of biotinylated horseradish peroxidase and streptavidin (SABC) was used for the detection of the bound primary antibody except for detection of the progesterone receptor where biotinylated swine-antirabbit immunoglobulin was used before SABC.

The scoring of the stained sections was done by using a semi-quantitative system based on the mean staining intensity and an estimation of the percentage of positive tumour cell nuclei (p53, cyclin D1, oestrogen and progesterone receptor) or percentage of cells with membrane staining of the tumour (HER2/neu, E-Cadherin). The mean staining intensity could vary from 0 (none), 1 (weak), 2 (moderate) to 3 (strong). The percentages of positive nuclei or membranes were categorized as 0 (0%), 1 (1% to 10%), 2 (11% to 25%), 3 (26% to 50%), 4 (51% to 75%) or 5 (76% to 100%). The staining score was calculated as the sum of mean staining intensity and percentage of positive tumour cells (range 0 to 8). p53 and cyclin D1 were analysed by using a staining score of 0 to 4 (called negative) and 5 or more (called positive). Expression of oestrogen and progesterone receptor, E-Cadherin and HER2/neu were analysed as any positive staining versus none. The cut-off levels used for scoring the immunohistochemical analyses of cyclin D1 and p53 were based on good correlation between immunohistochemistry and the presence of genetic alterations (cyclin D1, p53, E-cadherin and HER2/neu).

### *Procedure and availability of material*

All outcomes were assessed by the same observer: the clinical characteristics were assessed by HP; the histopathologic and molecular phenotypes were assessed by HP and MJ v d V. All data were gathered blinded for mutation status. Histological tissue was available of all 10 cases and 47 out of 50 control tumours. Regarding the patients receiving preoperative chemotherapy, all immunohistochemical-staining results were based on biopsies taken before the patients received this preoperative chemotherapy. Two of these patients showed a complete remission of their tumour. Consequently, for these patients tumour size was preoperatively radiological assessed, at the time of diagnosis, before neoadjuvant chemotherapy treatment, and DCIS, LCIS and lymphangio invasion could not be assessed. The radiological tumour size assessments were not included in the calculation of the mean tumour size.

### Statistical methods

Clinical and tumour characteristics were compared by using $\chi^2$ test or Fisher's exact test when any of the expected frequencies were less than five. We considered differences related to two-sided *P*-values less than .05 to be statistically significant. All analyses were performed using SPSS-PC version 9.0.

### RESULTS

Due to matching, the mean age at diagnosis of the 10 patients with *BRCA1* associated carcinomas and the 50 patients without *BRCA1* associated carcinomas was the same: 40.2 years (95% CI: 36.5 – 43.9 and 38.4 – 42.0, respectively), ranging from 30 to 48. Patients with BRCA1 associated carcinomas tended to have larger tumours and more often positive nodes (Table 1). Furthermore, the BRCA1 associated tumours tended to have a higher histologic grade, a less lower prevalence of in situ carcinoma, a lower frequency of high expression of cyclin D1 and a higher frequency of elevated p53 protein than the controls. None of these differences was statistically significant (Tables 2 and 3). Three patients received preoperative chemotherapy as part of a clinical trial (4 courses of 5-fluorouracil (600 mg/m$^2$), epirubicine (60 mg/m$^2$) and cyclophosphamide (600 mg/m$^2$)). BRCA1 associated tumours were more often oestrogen and progesterone receptor negative (respectively $P = 0.001$ and $P = 0.002$) (Table 3). No differences in the expression of HER2/neu and E-cadherin were observed between the two groups.

### DISCUSSION

The aim of this analysis was to study whether tumours of patients with *BRCA1* associated breast carcinomas are different from

© 2001 Cancer Research Campaign

**Table 1** Clinical characteristics in relation to mutation status

| | ***BRCA1*** **positive No. (%)** | ***BRCA1*** **negative No. (%)** | ***P*** |
|---|---|---|---|
| Tumour size (cm) | | | |
| – ≤ 2 | 3/10 (30) | 21/47 (45) | 0.49 |
| – > 2 | 7/10 (70) | 26/47 (55) | |
| – unknown | 0 | 3 | |
| – mean (SE) | 3.9 (0.91) | 3.1 (0.31) | |
| – range | 1.0 to 9.5 | 0.5 to 10.0 | |
| Axillary nodal status | | | |
| – negative | 3/9 (33) | 29/50 (58) | 0.28 |
| – positive | 6/9 (67) | 21/50 (42) | |
| – unknown | 1 | 0 | |
| Preoperative chemotherapy | | | |
| – yes | 3/10 (30) | 0/50 (0) | 0.004 |
| – no | 7/10 (70) | 50/50 (100) | |

**Table 2** Histologic tumour characteristics in relation to mutation status

| | ***BRCA1*** **positive No. (%)** | ***BRCA1*** **negative No. (%)** | ***P*** |
|---|---|---|---|
| Tumour type | | | |
| – ductal carcinoma | 8/10 (80) | 39/47 (83) | 0.92 |
| – lobular carcinoma | 1/10 (10) | 5/47 (11) | |
| – other | 1/10 (10) | 3/47 (6) | |
| Histologic grade | | | |
| – 1 | 0/10 (0) | 5/47 (11) | 0.24 |
| – 2 | 4/10 (40) | 26/47 (55) | |
| – 3 | 6/10 (60) | 16/47 (34) | |
| Mitotic rate | | | |
| – 1 (0–6) | 4/10 (40) | 26/47 (55) | 0.66 |
| – 2 (7–13) | 2/10 (20) | 8/47 (17) | |
| – 3 (>13) | 4/10 (40) | 13/47 (28) | |
| Tubule formation | | | |
| – 1 (> 75%) | 0/10 (0) | 5/47 (11) | 0.29 |
| – 2 (5–75%) | 1/10 (10) | 11/47 (23) | |
| – 3 (< 5%) | 9/10 (90) | 31/47 (66) | |
| Nuclear grade | | | |
| – I | 0/10 (0) | 0/47 (0) | 0.29 |
| – II | 2/10 (20) | 20/47 (43) | |
| – III | 8/10 (80) | 27/47 (57) | |
| DCIS# | | | |
| – absent | 6/8 (75) | 24/47 (51) | 0.27 |
| – present | 2/8 (25) | 23/47 (49) | |
| LCIS# | | | |
| – absent | 8/8 (100) | 41/47 (87) | 0.58 |
| – present | 0/8 (0) | 6/47 (13) | |
| Lymphangio invasion# | | | |
| – absent | 4/8 (50) | 37/47 (79) | 0.18 |
| – present | 4/8 (50) | 10/47 (21) | |

tumours of patients without *BRCA1* associated breast carcinomas in a hospital-based cohort not selected for family history or age at diagnosis. In *BRCA1* associated tumours receptors for oestrogen and progesterone were expressed less frequently than in controls. The finding regarding oestrogen and progesterone expression is in line with previous publications (Johannsson et al, 1997; Karp et al, 1997; Loman et al, 1998; Lynch et al, 1998; Verhoog et al, 1998; Armes et al, 1999). Absence of oestrogen and progesterone receptor expression has been even suggested as a potential indicator for the presence of a *BRCA1* germline mutation (Brown et al, 1999; Eisinger et al, 1999). In our study, the mutation carriers received preoperative chemotherapy more often ($P = 0.004$) than the patients in the control group. We consider this a chance difference because at the moment of treatment, a patient's *BRCA1* status was unknown.

**Table 3** Proportion of cases expressing immunohistochemical markers

| **Positive for** | ***BRCA1*** **positive No. (%)** | ***BRCA1*** **negative No. (%)** | ***P*** |
|---|---|---|---|
| Oestrogen receptor | 2/10 (20) | 32/41 (78) | 0.001 |
| Progesterone receptor | 2/10 (20) | 31/41 (76) | 0.002 |
| Cyclin D1 | 1/10 (10) | 15/43 (35) | 0.25 |
| p53 | 6/10 (60) | 11/42 (26) | 0.06 |
| HER2/neu | 1/10 (10) | 4/40 (10) | 1.0 |
| E-cadherin | 8/9 (89) | 34/40 (85) | 1.0 |

The trend towards a higher proportion of positives nodes among mutation carriers is supported by the study of Karp (Karp et al, 1997), whereas other groups did not find a difference (Armes et al, 1998; Verhoog et al, 1998), or reported a lower percentage of lymph node-positive cases among mutation carriers (Johannsson et al, 1997). The trend towards larger tumours among the mutation carriers was not reported by others (Johannsson et al, 1997; Armes et al, 1998; Loman et al, 1998; Verhoog et al, 1998). Some studies have reported that *BRCA1*-related breast tumours are more frequently of a medullary or atypical medullary type (Karp et al, 1997; Johannsson et al, 1997; Marcus et al, 1996; Armes et al, 1998; Lakhani et al, 1998), which we could not confirm. We did not find any differences regarding tumour type. In line with other studies (Eisinger et al, 1996; Marcus et al, 1996; Johannsson et al, 1997; Karp et al, 1997; Lakhani et al, 1997; Armes et al, 1998; Lynch et al, 1998), among the *BRCA1*-mutation carriers, a higher proportion of high-grade tumours with a higher mitotic rate, tubule formation and nuclear grade was observed. In a recent study in which differences between sporadic breast cancers and cancer involving *BRCA1* mutations were studied by means of a multifactorial model, a higher mitotic count has been identified as a key feature of the histologic phenotype of breast cancers in *BRCA1* mutations carriers (Lakhani et al, 1998). Also, in accordance with other studies (Lakhani et al, 1997; Armes et al, 1998), the *BRCA1*-related tumours were associated with a lower frequency of in situ carcinoma. The trend towards higher incidence of lymphangio invasion was not found by others (Armes et al, 1998; Robson et al, 1998). Armes et al, reported a significantly lower proportion of tumours showing cyclin D1 over-expression among *BRCA1*-related tumours (Armes et al, 1999). Our results showed the same differences, but did not reach statistical significance. The trend towards a higher proportion of p53-positive tumours among *BRCA1* carriers is in agreement with the results from several other studies which all reported statistically significant differences between mutation carriers and non-mutation carriers (Crook et al, 1997; Lynch et al, 1998; Armes et al, 1999; Philips et al, 1999). HER2/neu expression was infrequent and did not differ between the *BRCA1*-related tumours and the controls, which is in line with previous findings (Lynch et al, 1998). In accordance with previous publications concerning E-cadherin expression in *BRCA1* carriers (Robson et al, 1998; Armes et al, 1999; Jacquemier et al, 1999), we did not observe different expression levels.

As far as we know, this is the first study in which clinical and pathological features of *BRCA1* associated carcinomas are studied in a hospital-based cohort of breast cancer patients not selected for family history or age at diagnosis. The small number of

*© 2001 Cancer Research Campaign*

*BRCA1* mutation carriers in our cohort inherently limits the statistical power of the study so that for some variables only trends could be observed. Since the mutation analysis only would detect 70% of the mutations in the population, there may be an underestimation of the differences detected in the study. We conclude that the features of *BRCA1* associated tumours detected in a hospital-based series of breast cancer patients not selected for family history or age at diagnosis are similar to those tumours that were selected on these criteria. The opinion of the Breast Cancer Linkage Consortium that inclusion of breast-cancer histology may be part of the design of genetic testing policy (Lakhani et al, 1997; Lidereau et al, 2000) also seems to hold in a general population.

### ACKNOWLEDGEMENT

We would like to thank NJ Kuipers-Dijkshoorn for her valuable help with preparing the histological and immunohistochemical typing.

### REFERENCES

Armes JE, Egan AJ, Southey MC, Dite GS, McCredie MRE, Giles GG, Hopper JL and Venter DJ (1998) The histologic phenotypes of breast carcinoma occurring before age 40 years in women with and without *BRCA1* or *BRCA2* germline mutations: a population-based study. *Cancer* **83**: 2335–2345

Armes JE, Trute L, White D, Southey MC, Hammet F, Tesoriero A, Hutchins A-M, Dite GS, McCredie MRE, Giles GG, Hopper JL and Venter DJ (1999) Distinct molecular pathogeneses of early-onset breast cancers in *BRCA1* and *BRCA2* mutations carriers: a population-based study. *Cancer Research* **59**: 2011–2017

Brown DL, Cole BF and Arrick BA (1999) RE: Multifactorial analysis of differences between sporadic breast cancers and cancers involving *BRCA1* and *BRCA2* mutations. *J Natl Cancer Inst* **91**: 90–91

Cattoretti G, Pileri S, Parravicini C, Becker MH, Poggi S, Bifulco C, Key G, D'Amato L, Sabattini E and Feudale E (1993) Antigen unmasking on formalin-fixed, paraffin-embedded tissue sections. *J Pathol* **171**: 83–98

Crook T, Crossland S, Crompton MR, Osin P and Gusterson BA (1997) p 53 mutations in *BRCA1*-associated familial breast cancer. *Lancet* **350**: 638–639

Eisinger F, Stoppa-Lyonnet D, Longy M, Kerangueven F, Noguchi T, Bailly C, Vincent-Salomon A, Jacquemier J, Birnbaum J and Sobol HI (1996) Germline mutation at *BRCA1* affects the histoprognostic grade in hereditary breast cancer. *Cancer Res* **56**: 471–474

Eisinger F, Jacquemier J, Charafe-Jauffret E, Rio M-R, Birnbaum D and Sobol H (1999) More about: Multifactorial analysis of differences between sporadic breast cancers and cancers involving *BRCA1* and *BRCA2* mutations. *J Natl Cancer Inst* **91**: 1421–1422

Elston CW and Ellis IO (1991) Pathological prognostic factors in breast cancer. I. The value of histological grade in breast cancer: experience from a large study with long-term follow-up. *Histopathology* **19**: 403–410

Jacquemier J, Eisinger F, Nogues C, Sun ZZ, Guinebretiere JM, Peyrat JP, Geneix J, Lidereau R, Birnbaum D and Sobol H (1999) Histological type and syncytial growth pattern affect E-cadherin expression in a multifactorial analysis of a combined panel of sporadic and *BRCA1*-associated breast cancers. *Int J Cancer* **83**: 45–49

Johannsson OT, Idvall I, Anderson C, Borg A, Barakardóttir RB, Egilsson V and Olsson H (1997) Tumour biological features of *BRCA1*-induced breast and ovarian cancer. *Eur J Cancer* **33**: 362–371

Karp SE, Tonin PN, Bégin LR, Martinez JJ, Zhang JC, Pollak MN and Foulkes WD (1997) Influence of *BRCA1* mutations on nuclear grade and oestrogen receptor status of breast carcinoma in Ashkenazi Jewish women. *Cancer* **80**: 435–441

Lakhani SR (1997) Breast Cancer Linkage Consortium. Pathology of familial breast cancer: differences between breast cancers in carriers of *BRCA1* or *BRCA2* mutations and sporadic cases. *Lancet* **349**: 1505–1510

Lakhani SR, Jacquemier J, Sloane JP, Gusterson BA, Anderson TJ, Van de Vijver MJ, Farid LM, Venter D, Antoniou A, Storfer-Isser A, Smyth E, Steel CM, Haites N, Scott RJ, Goldgar D, Neuhausen S, Daly PA, Ormiston W, McManus R, Scherneck S, Ponder BA, Ford D, Peto J, Stoppa-Lyonnet D and Easton DF (1998) Multifactorial analysis of differences between sporadic breast cancers and cancers involving *BRCA1* and *BRCA2* mutations. *J Natl Cancer Inst* **90**: 1138–1145

Lidereau R, Eisinger F, Champeme MH, Nogues C, Bieche I, Birnbaum D, Pallud C, Jacquemier J and Sobol H (2000) Major improvement in the efficacy of *BRCA1* mutation screening using morphoclinical features of breast cancer. *Cancer Res* **60**: 1206–1210

Loman N, Johannsson O, Bendahl PO, Borg A, Ferno M and Olsson H (1998) Steroid receptors in hereditary breast carcinomas associated with BRCA1 or BRCA2 mutations or unknown susceptibility genes. *Cancer* **83**: 310–319

Lynch BJ, Holden JA, Buys SS, Neuhausen SL and Gaffney DK (1998) Pathobiologic characteristics of hereditary breast cancer. *Hum Pathol* **29**: 1140–1144

Marcus JN, Watson P, Page DL, Narod SA, Lenoir GM, Tonin P, Linder-Stephenson L, Salerno G, Conway TA and Lynch HT (1996) Hereditary breast cancer: pathobiology, prognosis, and *BRCA1* and *BRCA2* gene linkage. *Cancer* **77**: 697–709

Millar SA, Dykes DD and Polesky HF (1988) A simple salting out procedure for extracting DNA from human nucleated cells. *Nucleic Acids Res* **16**: 1215

Papelard H, De Bock GH, Van Eijk R, Vliet Vlieland TPM, Cornelisse CJ, Devilee P and Tollenaar RAEM (2000) Preyalence of *BRCA1* in a hospital-based population of Dutch breast cancer patients. *Br J Cancer* **83**: 719–724

Petrij-Bosch A, Peelen T, Van Vliet M, Van Eijk R, Olmer R, Drusedau M, Hogervorst FBL, Hageman S, Arts PJW, Ligtenberg MJL, Meijers-Heijboer H, Klijn JGM, Vasen HFA, Cornelisse CJ, Van't Veer LJ, Bakker E, Van Ommen G-JB and Devilee P (1997) *BRCA1* genomic deletions are major founder mutations in Dutch breast cancer patients. *Nat Genet* **17**: 341–345

Philips KA, Andrulise IL and Goodwin PJ (1999) Breast carcinomas arising in carriers of mutations in *BRCA1* or *BRCA2*: are they prognostic different? *J Clin Oncol* **17**: 3653–3663

Philips KA, Nichol K and Ozcelik H (1999) Frequency of p53 mutations in breast carcinomas of Ashkenazi Jewish *BRCA1* mutation carriers. *J Natl Cancer Inst* **91**: 469–473

Robson M, Gilewski T, Haas B, Levin D, Borgen P, Rajan P, Hirschaut Y, Pressman P, Rosen PP, Lesser ML, Norton L and Offit K (1998) BRCA-associated breast cancer in young women. *J Clin Oncol* **16**: 1642–1649

Shuber AP, Grondin VJ and Klinger KW (1995) A simplified procedure for developing multiplex PCRs. *Genome Res* **5**: 488–493

Van de Vijver MJ, Peterse JL, Mooi WJ, Wisman P, Lomans J, Dalesio O and Nusse R (1988) Neu-protein overexpression in breast cancer. Association with comedo-type ductal carcinoma in situ and limited prognostic value in stage II breast cancer. *N Engl J Med* **319**: 1239–1245

Verhoog LC, Brekelmans CT, Seynaeve C, Van den Bosch LMC, Dahmen G, Van Geel AN, Tilanus-Linthorst MMA, Bartels CCM, Wagner A, Van den Ouweland A, Devilee P, Meijers-Heijboer EJ and Klijn JGM (1998) Survival and tumour characteristics of breast-cancer patients with germline mutations of *BRCA1*. *Lancet* **351**: 316–321

*© 2001 Cancer Research Campaign*
